# Posttetanic potentiation improves neuromuscular efficiency of mouse muscle in vitro

**DOI:** 10.14814/phy2.15529

**Published:** 2022-12-02

**Authors:** Ryan Laidlaw, Rene Vandenboom

**Affiliations:** ^1^ Department of Kinesiology Faculty of Applied Health Sciences, Center of Bone and Muscle Health St. Catharine's Ontario Canada

**Keywords:** concentric, extensor digitorum longus, force, work

## Abstract

Neuromuscular efficiency is defined as the ratio of work output to stimulation rate. The purpose of these experiments was to test the hypothesis that neuromuscular efficiency would be increased in proportion to posttetanic potentiation, that is, the stimulation‐induced increase in work output displayed by rodent fast‐twitch muscle. To this end, extensor digitorum longus muscles from wild‐type and skeletal myosin light chain kinase knockout (skMLCK^−/−^) mice were surgically isolated and suspended in vitro (25°C). Concentric force development during shortening at 70% of maximal unloaded shortening velocity was tested at stimulus frequencies between 10 and 80 Hz both before and after a potentiating tetanus. A strong genotype‐dependent difference in the potentiation of concentric work output was observed; concentric work output of wild‐type muscles was increased by 51%–88% while that of skMLCK^−/−^ muscles was increased by only 20%–34% across the frequencies tested. As a result, comparison of work – frequency plots revealed that the frequency required for peak and 50% peak unpotentiated work of wild‐type muscles was decreased from ~80 to 52 Hz and from ~48 to 21 Hz, respectively. By contrast, the frequency required for peak and 50% peak unpotentiated work of skMLCK^−/−^ muscles was decreased from ~80 to 68 Hz and from ~51 to 41 Hz, respectively. Thus, wild‐type muscles with the ability to phosphorylate myosin displayed larger increases in neuromuscular efficiency than skMLCK^−/−^ muscles (25–30 vs 10–15 Hz, respectively). This suggests that the presence of myosin phosphorylation may ameliorate or mitigate fatigue mechanisms associated with high‐frequency stimulation rates.

## Graphical abstract

Posttetanic potentiation (PTP) is defined as the stimulation‐induced increase in muscle force, force, or power displayed by rodent fast‐twitch muscle. Our main result was that PTP increased neuromuscular efficiency to a greater degree in muscles from mice with the ability to phosphorylate myosin than without (in vitro, 25°C). This suggests that myosin phosphorylation may permit reductions in stimulus frequency to benefit mechanical performance of fast twitch skeletal muscle in vivo.

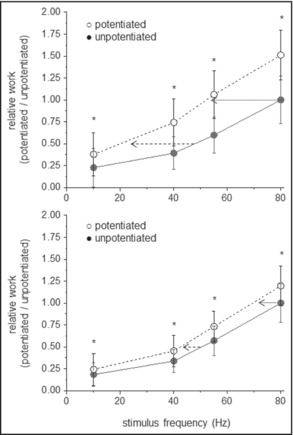

## 1 INTRODUCTION

Vertebrate skeletal muscle is a biological motor that transduces the chemical energy of adenosine triphosphate (ATP) to mechanical force, work, and power. A salient feature of fast‐twitch skeletal muscle is that force output may display considerable lability during long‐term performance. For example, prolonged activity may erode performance, a response described universally as fatigue (Allen et al., 2008). On the contrary, brief activity of fast fiber types has been shown to transiently enhance or “potentiate” force (Macintosh, 2003, 2010). Indeed, overall muscle performance may ultimately reflect the balance of these opposing processes (Krarup, 1981). Although the etiology of skeletal muscle fatigue is exceedingly complex, the primary mechanism for force potentiation is phosphorylation of myosin, as catalyzed by skeletal myosin light chain kinase (skMLCK) (reviewed by Stull et al., 2011). This reaction, which has been shown to increase the Ca^2+^ sensitivity of permeabilized skeletal muscles fibers (Persechini et al., 1985), is temporally correlated with force potentiation across a wide variety of fast‐twitch muscle models (see Vandenboom et al., 2013). Moreover, ablation of skMLCK and elimination of myosin phosphorylation greatly blunts, but does not entirely remove, force potentiation (Gittings et al., 2011; Zhi et al., 2005).

A surpassing aspect of muscle force potentiation is the ubiquitous nature of this phenomenon. For example, far from being restricted to rodents, potentiation is displayed by fast‐twitch muscles from other mammals including humans (reviewed by Blazevich & Babault, 2019). Indeed, both staircase potentiation, the gradual increase in isometric twitch force observed during low‐frequency stimulation (e.g., 5 Hz for 20 s), and posttetanic potentiation, the step increase in force observed after a brief high‐frequency volley (i.e., 100 Hz for 1 s), have been reported for various human skeletal muscles in vivo (e.g., Baudry & Duchateau, 2004, 2007; Desdmedt & Hainnaut, 1968; Houston et al., 1985; Vandervoort et al., 1983). Although the physiological significance remains to be ascertained, it is clear that human skeletal muscle, like fast‐twitch muscle from rodents, possesses the ability to potentiate under a wide variety of experimental conditions.

It has recently been suggested that myosin phosphorylation‐mediated forms of potentiation could, via direct influences on force output, increase neuromuscular efficiency in vivo (Zero & Rice, 2021). Neuromuscular efficiency is defined as the ratio of work output to stimulation rate (Tesch et al., 1990). Potentiation‐induced increases in neuromuscular efficiency could thus enhance locomotion by reducing the rate coding demand by working muscle. Although suggested by in vivo studies, this effect has not been quantified for isolated muscle. Thus, the purpose of this study was to assess how genotype‐dependent differences in posttetanic potentiation between wild‐type and skMLCK^−/−^ muscles would influence the stimulus frequency vs work relation in vitro. Genotype‐dependent differences in neuromuscular efficiency would provide a teleological explanation for the presence of myosin phosphorylation‐mediated forms of potentiation.

## 2 METHODS

### 2.1 Ethics statements and animal care

All research was conducted according to the Canadian Council on Animal Care (CCAC) guidelines. All animal housing and handling procedures received prior approval from the Brock University Animal Care Committee (protocol # 20–04‐01). Adult male wild‐type (C57BL/6) mice (≥ 10 weeks) were sourced from Charles River Laboratories (St. Constant, QC), while age‐matched skeletal myosin light chain kinase absent (skMLCK^−/−^) mice (male) entered the study from our breeding colony as needed. The masses of mice used in this study were similar between genotypes (22.2 ± 1.7 and 23.1 ± 2.0 g for skMLCK^−/−^ and wild‐type mice, respectively). Mice of both genotypes were housed according to guidelines set by the Good Animal Practice from the Canadian Council on Animal Care (CCAC). Mice were kept in a 14:10 light: dark cycle maintained at 20–22°C within an enriched environment. Standard mouse chow (5015, LabDiet, Aberfoyle, ON) and water were provided ad libitum. On the day of an experiment, mice were anesthetized via inhalation of isoflurane with a 2%–5% delivery rate and EDL muscles were surgically removed from the hindlimbs. Both muscles were incubated at just taut length (resting length) in an oxygenated bath containing cooled Tyrode's solution (5–10°C) until needed. Each muscle was eventually suspended vertically using noncompliant 4–0 silk suture in the 1200A intact muscle apparatus (Aurora Scientific Inc., Aurora, ON). The distal suture was clamped directly to the electrode assembly, and the proximal suture was secured to the servomotor arm via a short stainless‐steel wire. Contractile experiments were conducted at 25°C with the muscle immersed in continuously gassed (95% O_2_, 5% CO_2_) Tyrode's solution containing (in mM): 122 NaCl, 25 NaHCO_3_, 2.8 KCl, 1.2 KH_2_PO_4_, 1.2 MgSO_4_, 1.3 CaCl_2_, and 5.0 D‐glucose. An incubation period of at least 30–40 min allowed the muscles to equilibrate to the bath media, and then an automated protocol was performed to find optimal length (L_o_) for isometric twitch force. Muscles were stimulated using either single‐pulse or multiple‐pulse trains using a Model 701B high‐current stimulator (Aurora Scientific Inc., Aurora, ON). Stimulation intensity was set to ~1.25 times the voltage required for maximal twitch force to ensure that all muscle fibers were activated (≥ 60 V).

### 2.2 Experimental design

**FIGURE 1:**
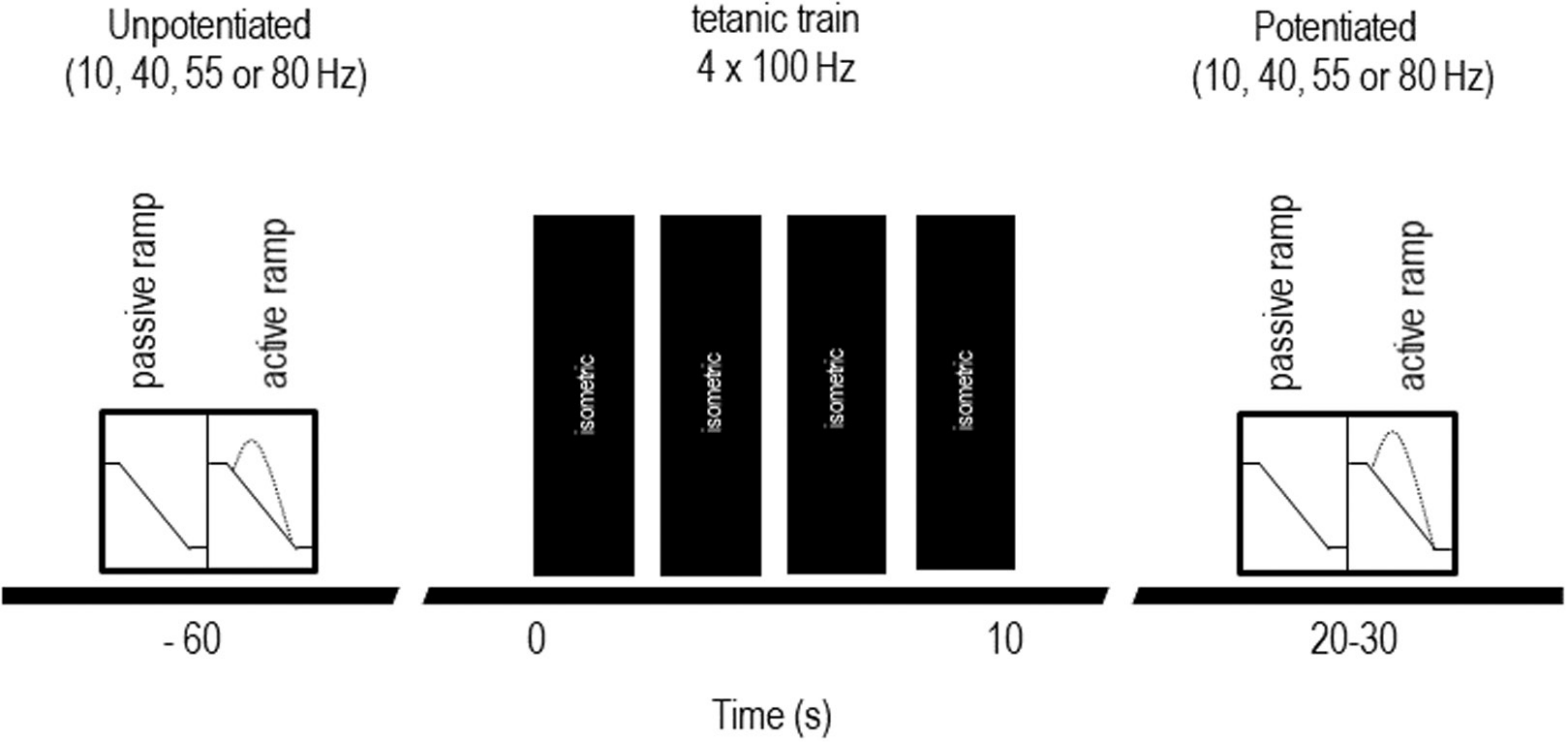
Schema showing experimental design testing the effect of tetanic stimulation on concentric force and work of wild‐type and skMLCK^−/−^ muscle (in vitro, 25°C). Concentric responses were obtained at one of the five test frequencies (i.e., 10, 40, 55, or 80 Hz) approximately 60 s before a tetanic train consisting of 4 volleys at 100 Hz within a 10 s span. Concentric responses were then repeated at the same test frequency ~ 25 s after the tetanic train. This bracketing procedure was repeated 4 times per muscle with ~20 min between tetanic trains. All concentric responses were obtained while shortening from 1.10 to 0.90 of optimal length (Lo) while shortening at 70% of maximal unloaded shortening velocity (Vmax). Note that during the tetanic train muscle length was held at Lo.

In this study, we compared unpotentiated (i.e., control) and potentiated (i.e., experimental) responses of muscles from two different mouse genotypes (wild‐type and skMLCK^−/−^). The experimental design is depicted in Figure 1. Each muscle of each genotype was stimulated to produce concentric forces at one of four different test frequencies (10, 40, 55 and 80 Hz) in randomized fashion. Unpotentiated trials were obtained ~1 minute before and potentiated trials were obtained ~20 s after a tetanic train, a time known to produce near‐maximal levels of potentiation in the mouse EDL muscle in vitro model (25°C) (see Vandenboom et al., 1995). The tetanic train consisted of 4 high‐frequency (100 Hz for 400 ms) volleys applied within a span of 10 s (with muscle held at Lo). The tetanic train protocol we used to induce potentiation has been shown to increase force, work, and power of mouse EDL muscle while inducing minimal levels of fatigue (Vandenboom et al., 1997; Xeni et al., 2011). A rest period of 20 min was allowed between adjacent protocols to allow potentiation to completely dissipate (Vandenboom et al., 1993). Comparison of post‐ to pretetanus responses (force and work) was used to determine the magnitude of potentiation for each test frequency (post/pre x 100).

### 2.3 Shortening and stimulation protocol

**FIGURE 2:**
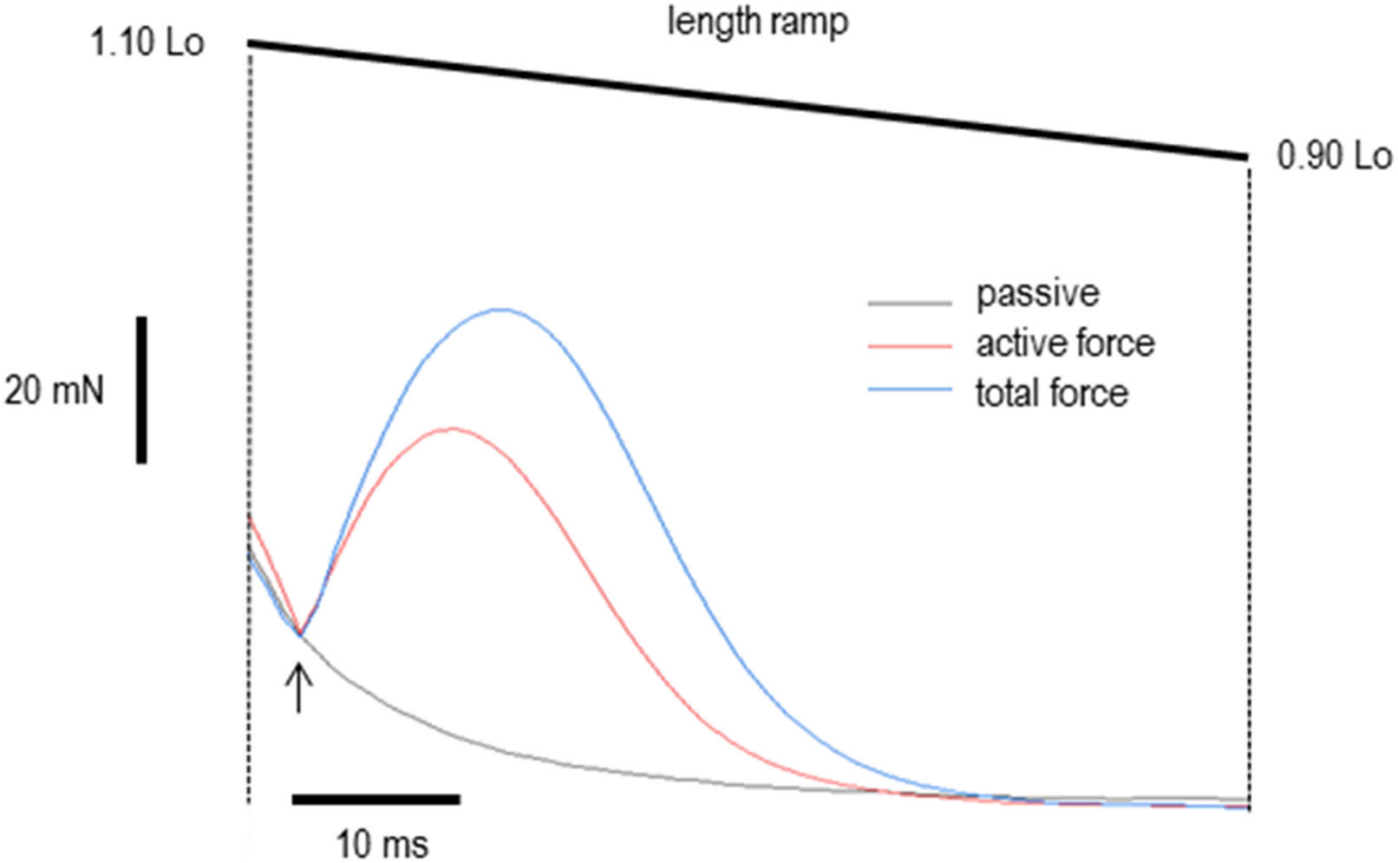
Example of passive, total, and active concentric force for 10 Hz trials before and after the tetanic train (superimposed). Note that at this frequency and shortening speed only one pulse was delivered during the available time window. Paired trials incorporating length changes took place before and after the tetanic train. Length change amplitude and speed were identical for all trials (shown at top). During the passive trial, the muscle shortened but was not stimulated; this produced only a change in passive tension (gray line). During the active trial, the muscle was stimulated (arrow), producing a recording of total force (blue). When the passive force was digitally subtracted from the total force, a record representing active twitch force was produced (red). The active force record was used to determine peak and mean concentric force for each trial and each frequency. Similar procedures were used for each frequency. Vertical bar represents force scale, while horizontal bar represents time scale. Vertical dashed lines represent start and end of length ramp.

An example of the shortening protocol is shown in Figure 2 for the 10 Hz frequency. During each test, contraction muscles shortened from 1.10 to 0.90 Lo at a constant rate of ~6.9 muscle lengths per second; this was equivalent to 0.70 of maximal shortening velocity for mouse EDL muscles (both genotypes) at 25°C (Gittings et al., 2011). Stimulation rate varied from protocol to protocol but stimuli always had the same starting time point during the down ramp. Because ramp duration was constant for each muscle, the number of stimuli, and thus force pulses, varied from protocol to protocol (10 Hz, 40 Hz, 55 Hz, and 80 Hz stimulation produced 1, 2, 3, and 4 pulses, respectively). In all cases, the calculation of mean concentric force and work was restricted to the period of shortening only.

### 2.4 Analysis of concentric force and work

Active concentric force for each frequency and condition was calculated as the difference between total force and the corresponding passive force obtained during the paired stimulated and passive ramps, respectively. An example of this method is shown in Figure 2. The method was the same for unpotentiated and potentiated trials and for each frequency. A “passive” trial was always paired (5 s apart) with a “stimulated trial” with the difference being that the muscle was stimulated during the latter and not the former. As a result, the two trials produced corresponding force records reporting passive and total force, respectively. The passive force record was digitally subtracted from the total force record to produce a record of “active force” for each trial in each condition and for each frequency. Custom software was then used to determine mean concentric force during each trial (mN). Muscle work output was determined by multiplying mean concentric force (mN) by the corresponding length change (mm). This value was normalized to muscle mass and expressed as J kg^−1^. Because the rate and amplitude of shortening was constant for all frequencies, total work output was a direct assay of mean force output in the unpotentiated and potentiated states. To determine neuromuscular efficiency, we compared the work versus stimulus frequency relationship for potentiated and unpotentiated conditions; this comparison allowed us to determine the reduction in stimulus frequency required for peak and subpeak work out in the potentiated vs unpotentiated state for each genotype.

### 2.5 Statistical analysis

The effect of the conditioning stimulus on absolute contractile values was investigated with two‐tailed paired Student's *t*‐tests at each frequency for each genotype. To compare relative force across genotypes, a 2 (genotype) by 4 (frequency) factorial ANOVA was conducted where frequency was a within‐group variable and genotype a between‐group factor. The results of Mauchly's test (W (5) = 0.59, *p* > 0.05) and Box's test (M (10, 1912.35) = 19.47, *p* > 0.05) showed that the data upheld the assumptions of sphericity and equality of covariance matrices, respectively. All analyses were conducted using IBM SPSS Statistics version 28.0.0.0.

## 3 RESULTS

### 3.1 Concentric responses

**FIGURE 3:**
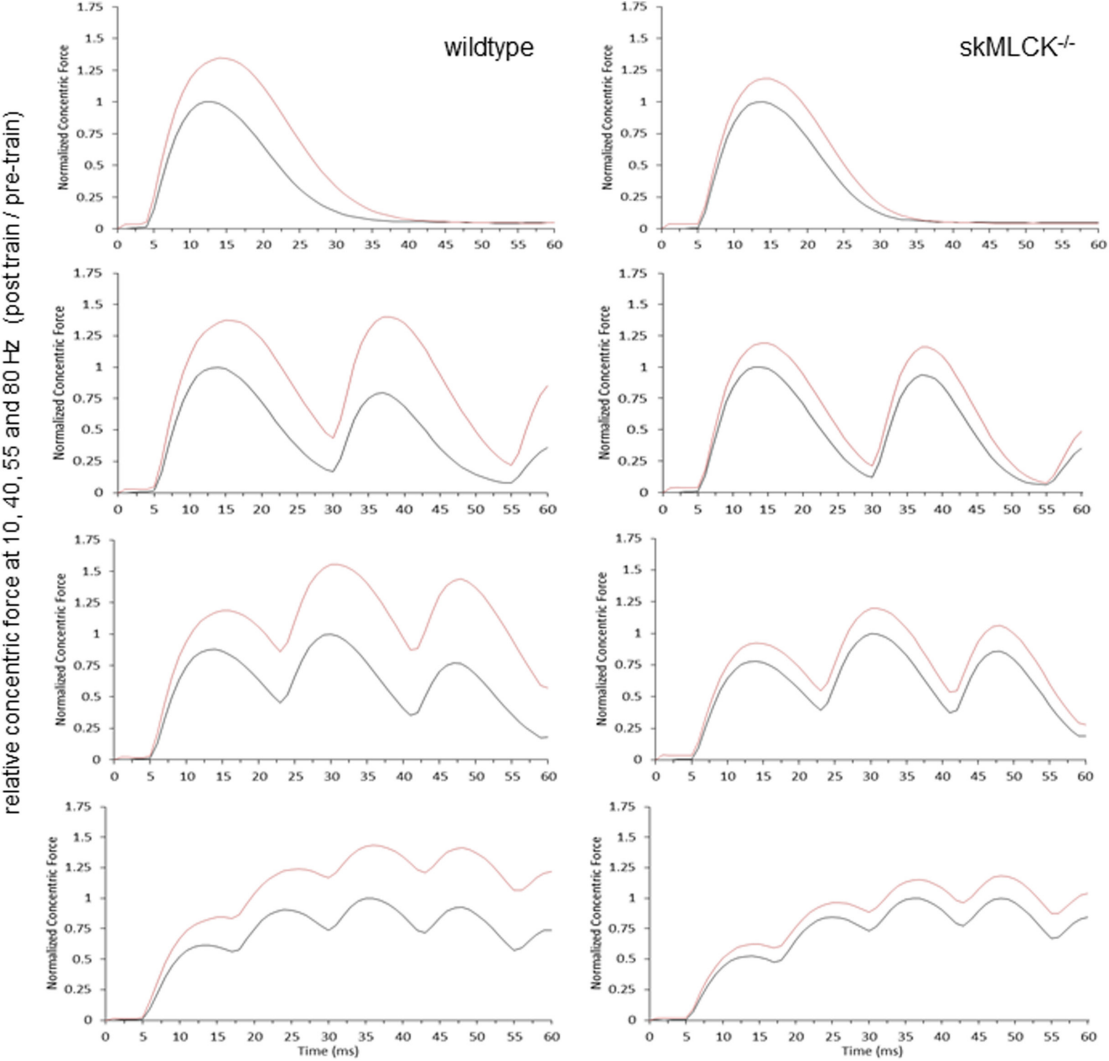
Representative active concentric force records at select test frequencies for wild‐type (left) and skMLCK^−/−^ (right) muscles. From top to bottom: 10 Hz, 40 Hz, 55 Hz, and 80 Hz. Black trace is unpotentiated, and red trace is potentiated record. The amplitude of each record for each genotype is normalized to the unpotentiated value at that frequency. Records are truncated at the end of isovelocity shortening from 1.10 to 0.90 Lo at 0.70 Vmax. Note the increase in pulse amplitude at every frequency for wild‐type, and to a much lesser extent, skMLCK^−/−^ muscles.

Representative records illustrating the effect of the tetanic train on active concentric force are shown in Figure 3 with results from all experiments summarized in Tables 1 and 2, respectively. These data reveal that although unpotentiated concentric forces were similar between genotypes at every frequency, wild‐type muscles displayed a much greater increase at every frequency than did skMLCK^−/−^ muscles, that is, there was a genotype‐dependent difference in concentric force potentiation. Because shortening amplitude was constant for all conditions, the increase in peak concentric force observed produced large increases in the work performed by wild‐type, and to a lesser extent, skMLCK^−/−^ muscles at each frequency tested. In relative terms, the potentiating stimulus increased the work performed by wild‐type and skMLCK^−/−^ muscles by 51–89 and by 20%–34% across the different frequencies tested, respectively.

**TABLE 1:** Effect of tetanic protocol on concentric force at different frequencies

		Stimulus frequency			
Genotype	Condition	10 Hz	40 Hz	55 Hz	80 Hz
wild Type	Pretetanic	14.2 ± 2.60	13.7 ± 2.43	15.9 ± 3.90	22.4 ± 6.49
	Posttetanic	19.4 ± 3.36^a^	20.0 ± 3.93^a^	24.9 ± 5.76^a^	32.4 ± 7.96^a^
	Relative	1.37 ± 0.10^b^	1.46 ± 0.17^b^	1.57 ± 0.10^b^	1.45 ± 0.10^b^
skMLCK^−/−^	Pretetanic	12.4 ± 3.86	12.9 ± 3.36	16.5 ± 4.66	24.4 ± 7.22
	Posttetanic	14.8 ± 4.30^a^	15.0 ± 4.06^a^	19.7 ± 5.36^a^	28.4 ± 8.66^a^
	Relative	1.19 ± 0.07	1.19 ± 0.03	1.19 ± 0.07	1.16 ± 0.10

*Note*: Values are peak force in mN (mean ± *SD*, *n* = 12). Pre‐ or unpotentiated values were obtained before while post or potentiated values were obtained 20 s after the tetanic train, respectively. Relative values are potentiated divided by unpotentiated for that frequency and genotype.

^a^ Potentiated value greater than unpotentiated value for that frequency and genotype (*p* < 0.05).

^b^ Wild‐type value greater than respective skMLCK^−/−^ value (*p* < 0.001).

**TABLE 2:** Effect of tetanic protocol on work output at different frequencies

		Stimulation frequency			
Genotype	Condition	10 Hz	40 Hz	55 Hz	80 Hz
Wild type	Pretetanic	0.55 ± 0.17	0.90 ± 0.30	1.37 ± 0.53	2.29 ± 0.93
	Posttetanic	0.90 ± 0.27^a^	1.70 ± 0.57^a^	2.44 ± 0.83^a^	3.46 ± 1.20^a^
	Relative	1.64 ± 0.13^b^	1.88 ± 0.13^b^	1.78 ± 0.13^b^	1.51 ± 0.17^b^
skMLCK^−/−^	Pretetanic	0.51 ± 0.0.13	0.93 ± 0.27	1.57 ± 0.53	2.75 ± 0.90
	Posttetanic	0.67 ± 0.20^a^	1.25 ± 0.37^a^	2.01 ± 0.57^a^	3.29 ± 0.93^a^
	Relative	1.31 ± 0.10	1.34 ± 0.13	1.28 ± 0.13	1.20 ± 0.13

*Note*: Values are work in j kg^−1^ (mean ± *SD*, *n* = 12 each genotype). Pre‐ or unpotentiated values were obtained before while post or potentiated values were obtained 20 s after the tetanic train, respectively. Work was calculated over the force‐producing period of the concentric phase of each contraction while shortening from 1.10 to 0.90 L_o_ at 0.70 Vmax. Relative values are potentiated divided by unpotentiated for that frequency and genotype.

^a^ Potentiated value greater than unpotentiated value for that frequency and genotype (*p* < 0.05).

^b^ Wild‐type value greater than respective skMLCK^−/−^ value (*p* < 0.001).

### 3.2 Neuromuscular efficiency

**FIGURE 4:**
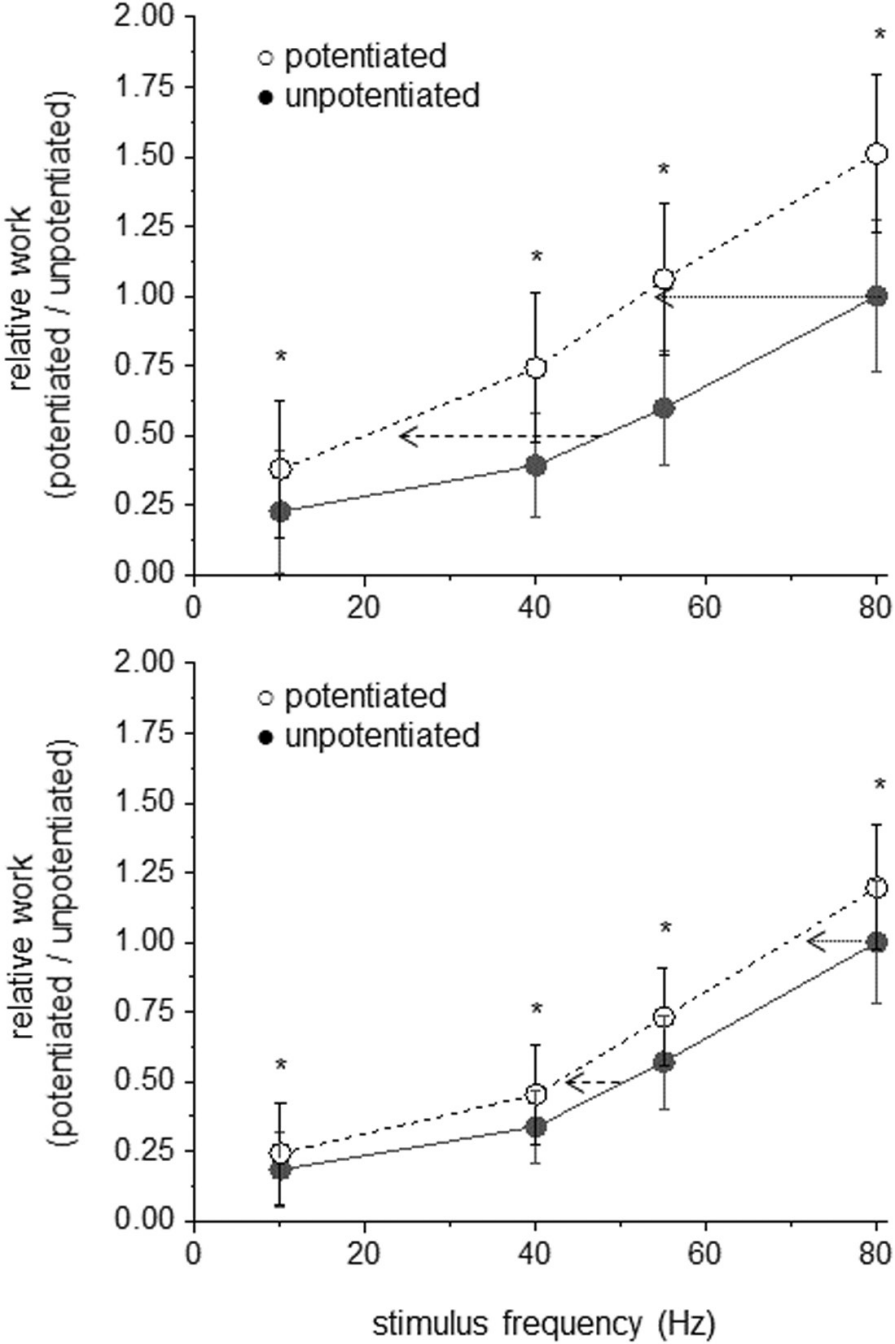
Effect of potentiation on neuromuscular efficiency of wild‐type and skMLCK^−/−^ muscles. Panels show effect of tetanic train on relative work of wild‐type (top) skMLCK^−/−^ (bottom) muscles. In each panel, curve with solid symbols is unpotentiated work scaled to value at 80 Hz (1.00) while curve with open symbols is potentiated work expressed relative to unpotentiated (potentiated/unpotentiated). Horizontal arrows depict reduction in stimulus frequency required for 100 (dotted) and 50% (dashed) of peak work output in potentiated relative to unpotentiated state for each genotype. The increase in work caused by the tetanic train was greater for wild‐types than for skMLCK^−/−^ muscles at each frequency. *Potentiated value significantly greater than unpotentiated value at that stimulus frequency (*p* < 0.05). Data are mean +/− *SD*.

The effect of potentiation on the work versus frequency relationship for both genotypes is shown in Figure 4. For wild‐type muscles, the stimulus frequency required to duplicate 100 or 50% of unpotentiated work was reduced from 80 to ~52 Hz and from 48 to ~21 Hz, respectively. On the contrary, the stimulus frequency required to duplicate 100 or 50% of unpotentiated work was reduced from 80 to 68 Hz and from 51 to 41 Hz in skMLCK^−/−^ muscles. As a result, although increased in both genotypes, the effect of potentiation on apparent neuromuscular efficiency was markedly greater for wild‐type than skMLCK^−/−^ muscles. The reason for this effect was the large calculated increase in the work performed per stimulus pulse, an effect that was approximately 2 x greater for wild‐type than for skMLCK^−/−^ muscles at each frequency.

## 4 DISCUSSION

The purpose of these experiments was to test the hypothesis that neuromuscular efficiency would be increased in proportion to genotype‐dependent differences in posttetanic potentiation displayed by wild‐type and skMLCK^−/−^ muscles. Our main findings were that the potentiation of work displayed by wild‐type muscles decreased the stimulus frequency required to produce peak and subpeak work by 25–30 Hz. On the contrary, the lesser potentiation of work displayed by skMLCK^−/−^ muscles decreased the stimulus frequency required to produce peak and subpeak work by 10–15 Hz, respectively. The proportional decrease in the rate coding required for work with increasing potentiation is indicative of a functional relationship. Hence, although both genotypes benefited from potentiation our results indicate that myosin phosphorylation greatly amplifies this effect. This result has important implications for improving our understanding of how force potentiation may be accommodated by neuromotor control of fast‐twitch skeletal muscle in vivo.

Our work is one of many showing a positive effect of potentiation on work output of rodent striated skeletal muscle, first demonstrated by Grange et al. (1995, 1998). Myosin phosphorylation has been correlated with the rate of isometric force development of mouse EDL muscle (e.g., Vandenboom et al., 1995, 1997), an effect that may also enhance concentric force and work when shortening is allowed. Indeed, inspection of the force records in Figure 3 reveals that wild‐type muscles displayed a much greater rate of rise of force in the potentiated state. Although unclear, a possible mechanism for the relatively small magnitude of work potentiation displayed by skMLCK^−/−^ muscles in our experiments is a tetanic‐induced elevation in resting myoplasmic calcium levels (see Smith et al., 2013). If so, it is likely that this mechanism also contributed to the work potentiation displayed by wild‐type muscles; as a result, the genotype‐dependent difference in work we report accords with genotype‐dependent differences in myosin phosphorylation. Potential candidates for myosin phosphorylation‐independent forms of potentiation have been discussed recently (Angelidis et al., 2022; Overgaard et al., 2022).

Far from being restricted to rodents, force potentiation phenomena may be a ubiquitous feature of muscle performance in nature. For example, potentiation has been observed in muscles or muscle fibers from such diverse species as birds (Taylor‐Burt et al., 2020), cats (Brown & Loeb, 1998), dogs (Martin‐Flores et al., 2011), frogs (Vergara et al., 1977), insects (Dickinson et al., 1997), and spiders (Padrón et al., 2020). Based on this, it is difficult to argue that potentiation mechanisms are not highly relevant for the locomotion of animals and humans. For example, given that pulse number and not pulse frequency may be the primary regulated variable during locomotion (Hennig & Lømo, 1985; Hennig & Lømo, 1987), potentiation‐mediated increases in work per pulse may help reduce rate coding requirements. This idea explains observations of a reduced motor unit firing rate during voluntary contractions of human muscle either in the presence (Adam & De Luca, 2005; Bigland‐Ritchie et al., 1983; Dorfman et al., 1990; Suzuki et al., 1988; Woods et al., 1983) or absence of fatigue (Inglis et al., 2011; Klein et al., 2001). Although these studies all used isometric contractions, their findings may also be applicable to dynamic contractions.

A novel aspect of our work was the high shortening speed and activation rates at which concentric potentiation was observed (i.e., 0.70 Vmax and 80 Hz). Previous work from our laboratory has demonstrated that the potentiation of concentric responses is both shortening speed – and stimulus frequency—dependent in the mouse EDL muscle model (Caterini et al., 2011; Gittings et al., 2012). Although our results suggest a saturation of this effect at speeds above 0.50 Vmax, it seems apparent that substantial potentiation of concentric force may be evident at high frequencies during fast shortening (i.e., 0.25–0.75 Vmax). This interaction may be relevant for the intact human neuromuscular system where motor unit discharge rates of 115–120 Hz have been reported for ballistic movements (Desmedt & Godaux, 1977, 1978; Van Cutsem et al., 1998; Van Cutsem & Duchateau, 2005). Although these high motor unit discharge rates may not be constant (Enoka & Duchateau, 2017), they may still pose limitations on neuromuscular function. For example, at the single fiber level, isometric force declines during high‐frequency stimulation (100 Hz) have been attributed to action potential propagation failure in the t‐tubules (Duty & Allen, 1994; Westerblad et al., 1990). Under these experimental conditions, reductions in stimulation rate lead to a recovery of force. Based on this, potentiation‐induced increases in neuromuscular efficiency that allow for reductions in motor unit discharge to occur may provide a similar benefit.

In the present work, we tested the concentric force response at a range of stimulus frequencies that may be considered physiological while the muscle was allowed to shorten at near‐maximal rates. This design may relegate the general applicability of our results, however (but see above). Although our experiments were conducted at 25°C, a temperature that could be considered the lower limit for mammalian skeletal muscle, the potentiation of isometric force of mouse EDL muscle is inversely related to temperature in the range 25–35°C (Moore et al., 1990). Whether this is also true for concentric force potentiation remains to be determined but leaves open the possibility that our results may actually underestimate the influence of potentiation on neuromuscular efficiency. Finally, although not measured in the current work, previous studies from our laboratory have quantified genotype‐dependent differences in myosin phosphorylation (e.g., Bowslaugh et al., 2016; Bunda et al., 2018; Gittings et al., 2011).

In summary, although not a surrogate for the intact neuromuscular system, results from our in vitro muscle model may provide some insights into the reduced motor unit discharge rate displayed by human skeletal muscle during potentiation. Although the afferent signal pathway and/or integration remains obscure, potentiation‐induced increases in neuromuscular efficiency would allow equivalent submaximal work output concomitant with reduced motor unit discharge rate.

## FUNDING INFORMATION

Work was funded by the National Science Engineering Research Council (Canada) (RV). Grant number: 2019–05122. Grant title: Estrogen, Myosin Phosphorylation and Muscle Thermogenesis.

## CONFLICT OF INTEREST

The authors (RL and RV) have no competing interests or financial or nonfinancial interests that are directly or indirectly related to the work submitted for publication.

## DATA AVAILIBILITY STATEMENT

Data can be made available upon reasonable request to the corresponding author (RV).
